# A prospective, multicenter, clinical study of duodenoscope contamination after reprocessing

**DOI:** 10.1017/ice.2021.525

**Published:** 2022-12

**Authors:** Naoaki Okamoto, Anna Sczaniecka, Misa Hirano, Melinda Benedict, Shigeyoshi Baba, Yoko Horino, Masato Takenouchi, Yuichi Morizane, Ikuo Yana, Ross Segan, Lionel Pineau, Michelle J. Alfa

**Affiliations:** 1 Medical & Scientific Affairs, Olympus Corporation, Hachioji, Tokyo, Japan; 2 Gyrus ACMI, (Olympus Surgical Technologies America), Redmond, Washington; 3 Medical & Scientific Affairs, Olympus Corporation of the Americas, Center Valley, Pennsylvania; 4 Eurofins Biotech Germande France, Marseille, France; 5 Department of Medical Microbiology, University of Manitoba, Winnipeg, Manitoba, Canada

## Abstract

**Objective:**

Several clinical procedures utilize duodenoscopes, which are processed for reuse after the procedures are completed. However, infection outbreaks due to improper duodenoscope processing occur frequently. To address this, we aimed to assess the contamination rates of duodenoscopes after reprocessing in nonoutbreak settings.

**Design and setting:**

Prospective study in 16 clinical sites in the United States.

**Methods:**

We sampled and cultured reprocessed duodenoscopes following the FDA/CDC/ASM guideline; “Duodenoscope Surveillance Sampling and Culturing – Reducing the Risks of Infection.” High-concern (HC) organisms were those highly associated with disease, including gram-negative rods, *Staphylococcus aureus, Staphylococcus lugdunensis*, β-hemolytic *Streptococcus*, *Enterococcus* spp, and yeasts. We evaluated duodenoscopes with ≥1 CFU of organisms after reprocessing. The reprocessing environments were also sampled and cultured.

**Results:**

We assessed 859 newer-model (NM) duodenoscopes (TJF-Q180V) and 850 older-model (OM) duodenoscopes (TJF-160F/VF); of these, 35 NM samples (4.1%) and 56 OM samples (6.6%) were contaminated with HC organisms. We detected and classified the HC organisms as gastrointestinal (45.4%), human origin (16.7%), environmental (24.1%), waterborne (13.0%), and unidentified (0.9%).

**Conclusions:**

We detected an overall HC contamination rate of 5.3% in nonoutbreak settings. Although the relationship between endoscopic contamination and the occurrence of infections remains unclear, attempts should continue to be made to further reduce contamination rates. Additional improvements to the manufacturer’s instructions for use, human factors during the reprocessing procedure, ongoing training programs, cleanliness of reprocessing environments, and the design of the distal end of the duodenoscope should be considered.

Introduced in 1968, endoscopic retrograde cholangiopancreatography (ERCP) is an advanced medical procedure meant to carry out a minimally invasive diagnosis and manage pancreaticobiliary conditions using duodenoscopes. More than 650,000 patients undergo ERCP in the United States annually.^1,2^

According to Spaulding’s classification, duodenoscopes are semicritical, nonsterile devices that require high-level disinfection (HLD). ERCP is customarily performed in endoscopy rooms, which are nonsterile environments. Therefore, during ERCP, duodenoscopes should be free of all microorganisms, except low concentrations of bacterial spores.

Endoscopy-related infections can be endogenous or exogenous.^3^ Most postendoscopy infections are endogenous, that is, they arise from the patient’s microbiota and they cannot be prevented by endoscope reprocessing or sterilization. Exogenous infections arise from contaminated endoscopes and constitute a minority of duodenoscope-associated infections.^2^ The incidence of infectious complications following ERCP is 2%–4%,^3^ with pancreatitis being the most common post-ERCP complication.^4^

An analysis of endoscope-related medical device reports during 1997–2015 identified 146 cases of duodenoscope-related infections.^5^ Most reports were submitted during 2010–2015, and ∼3 million ERCP procedures were performed.^5^ Balan et al^1^ performed a systematic search of MEDLINE and EMBASE databases (2008–2018) and identified 490 infected patients among 24 clusters worldwide. The number of infected patients peaked sharply in 2012 and plummeted thereafter.^1^ The likelihood of endoscopy-associated infections is often referenced from a 1993 paper that reported the risk of 1 exogenous infection for every 1.8 million endoscopies (0.00006%).^6^ Another study^7^ stated that this infection rate might be underestimated, and another^8^ reported that it is “inaccurate and outdated.”

The rate of exogenous infection from contaminated endoscopes is currently unknown. Duodenoscopes contaminated with certain microorganisms may cause infection, but “contamination” (presence of microorganisms on a duodenoscope) and “infection” (microorganisms invading the patient tissue or causing clinical disease) are not synonymous. Not all organisms are pathogenic; the infection frequency and impact of each contaminating organism are unclear. The incidence rates of duodenoscope contamination vary greatly (0.4–30%).^9–14^ Limited data suggest that 12%–41% of patients exposed to a contaminated duodenoscope during an outbreak develop infections^15^; however, this does not represent the transmission in a nonoutbreak setting. Endoscope contamination outbreaks have been associated with multifactorial etiologies, including reprocessing protocol breaches,^16^ inadequate endoscope maintenance,^17^ duodenoscope design issues,^18^ and ineffective or absent microbiological surveillance.^15,19^ Delayed precleaning, inadequate cleaning and drying, and faulty scope design may also cause contamination.^2^ The US Food and Drug Administration (FDA) has published guidelines for using duodenoscopes in addition to the standard HLD protocol for duodenoscope reprocessing, including surveillance cultures of patient-ready endoscopes after HLD.^20^ Device manufacturers have also updated reprocessing protocols, including the use of FDA-approved brushes and supplemental flushing measures to address areas difficult to clean, such as the “elevator recess.”

On October 5, 2015, after examining patients with carbapenem-resistant *Enterobacteriaceae* infections following ERCP,^21^ the FDA ordered all duodenoscope manufacturers to conduct postmarket surveillance (PMS) studies to assess duodenoscope contamination rates following HLD and to identify the factors causing duodenoscope contamination. As part of these PMS studies, we investigated the duodenoscope contamination rate by viable microorganisms after following the manufacturer’s FDA-cleared reprocessing instructions.

## Methods

### Sample collection

According to the FDA PMS study directive, we included duodenoscope models TJF-Q180V (newer) and TJF-160F/VF (older). Of the 33 invited US clinical sites, 16 contributed to this study: 10 hospital-based endoscopy suites (ie, large facilities) and 6 ambulatory surgical centers (ie, small healthcare providers). Contracts were executed with all facilities before the study, and institutional review board approval was obtained at the facilities as necessary. After bedside precleaning, manual cleaning, and automated reprocessing, including HLD, trained individuals sampled the duodenoscopes using a centrally distributed sample collection kit (OLYMPUS assembled, Center Valley, PA). We followed methods outlined by the FDA, Centers for Disease Control and Prevention (CDC), and the American Society for Microbiology (ASM).^22^ The sampling staff wore bouffant caps, face masks, sterile gowns, and sterile gloves, and they handled the samples in a field prepped with a sterile surgical drape. We sampled the instrument channel (biopsy port to the distal end) using the flush–brush–flush method with sterile water and a sterile wire-shaft bristle brush. A swab sample of the distal exterior surface and distal tip face was collected. The flush–brush–flush method was then used to collect samples from the elevator recess cavity.

Fluid samples from the instrument channel, the tip of the swab, and bristled head of the brush used for sampling were collected in the same container. Sterile Dey-Engley broth (General Laboratory Products, Yorkville, IL) was added to the sample in a 1:1 ratio as a neutralizing solution. The samples were sealed, packaged with ice packs, and transported to a laboratory for culturing.

The duodenoscopes were quarantined until the cultures were negative for “actionable” organisms (according to the FDA, CDC, and ASM protocols).^22^ Duodenoscopes with “actionable” organisms were sent for destructive evaluation (results not presented).

### Culture and identification of microorganisms

The sample containers were maintained at a temperature of 2–8 ± 2°C until they were processed at the laboratory (NAMSA, Irvine, CA) within 32 hours after collection at the sites. A validation test for sample transport was completed prior to the study. After spinning the container in a vortexer, the swab and brush heads were aseptically removed using sterile forceps. The sample was filtered through a 0.45-μm filter that was placed on a blood agar plate. Bacterial growth (in colony-forming units or CFU) was quantified after 72 hours of incubation at 35–37°C. The limit of detection for this culture method was 1 CFU per endoscope sample.

If the culture plate remained negative at 72 hours of incubation, the duodenoscope was released from quarantine for further use. Colonies in positive-culture plates were removed and tested by Gram stain, and the colonial and cellular morphologies were documented. The colonies were subcultured, and the species were identified using 16s rRNA gene sequencing on the MicroSEQ ID Microbial Identification System (Applied Biosystems, Waltham, MA).

### Definition of microorganism and contamination

The identified microorganisms were classified as low- to moderate-concern organisms (LMC) and high-concern (HC) organisms according to FDA–CDC–ASM surveillance protocols,^22^ which state that, compared with other organisms, HC organisms are often associated with disease. HC organisms included gram-negative organisms (eg, *Escherichia coli, Klebsiella pneumoniae, Enterobacteriaceae,* and *Pseudomonas aeruginosa*) and gram-positive organisms (eg, *Staphylococcus aureus, Staphylococcus lugdunensis*, β-hemolytic *Streptococcus, Enterococcus* spp, and yeasts). As a precaution, we defined all gram-negative rods as HC organisms, including potentially opportunistic organisms (usually pathogenic only to plants but may infect humans under unique circumstances). We classified HC organisms into 4 categories: gastrointestinal, human origin (other than gastrointestinal), environmental, or waterborne.

The LMC organisms included filamentous fungi, several gram-positive bacterial species (eg, *Micrococcus*, coagulase-negative staphylococci (excluding *S. lugdunensis*), *Bacillus*), and diphtheroids or other gram-positive bacilli. Organisms of moderate concern comprise oral-cavity colonizers (eg, saprophytic *Neisseria*, viridans group streptococci, and *Moraxella* spp). We classified LMC organisms into 2 categories: human origin or environmental.

Endoscope contamination was defined as “actionable” per the FDA–CDC–ASM protocols^22^ as follows: (1) growth of ≥1 CFU of any HC organism on a cultured plate or (2) growth of >100 CFU of any LMC organism on a cultured plate.

### Technical review, environmental assessment, and sampling

If contamination was detected, sampling and reprocessing at the concerned collection site were reviewed by a trained specialist using a designated check sheet to identify instances of improper handling.

We also conducted environmental culturing to assess the role of accidental contamination from the duodenoscope sampling and reprocessing areas. These samples were acquired by swabbing up to 20 sampling points (eg, floor, sink drain, automated endoscope reprocessor (AER) lid, touch panel of AER, and others) in relevant areas (eg, sampling area, AER, reprocessing room, and others).

To identify contamination not of patient origin, negative control samples were collected from duodenoscopes that underwent ethylene oxide (EtO) sterilization and subsequent AER HLD and from duodenoscopes used in the clinical study that were EtO sterilized but did not undergo AER to evaluate whether the AER affected the negative control-culture results. Negative control samples were only collected from TJF-Q180V duodenoscopes.

## Results

Between October 2018 and September 2019, we obtained 859 and 850 samples from TJF-Q180V and TJF-160F/VF duodenoscopes, respectively, at 16 collection centers. Overall, 91 samples from both scope models were contaminated by HC organisms, with a contamination rate of 5.3%. Additionally, 13 samples from both models were contaminated by >100 CFU LMC organisms, with a contamination rate of 0.8% (Table 1). Of the duodenoscopes cultured, 34.8% demonstrated no detectable CFU (Table 1).

**Table 1.** Culture Results of Different Olympus TJF-Q180V and TJF-160F/VF Duodenoscope Models

Cultures Collected	TJF-Q180V (n=859), No. (%)	TJF-160F/VF (n=850), No. (%)	Total (n=1,709), No. (%)
HC organisms	35 (4.1)	56 (6.6)	91 (5.3)
>100 CFU of LMC organisms	3 (0.3)	10 (1.2)	13 (0.8)
11–100 CFU of LMC organisms	49 (5.7)	33 (3.9)	82 (4.8)
1–10 CFU of LMC organisms	473 (55.1)	456 (53.6)	929 (54.3)
0 CFU no contamination	299 (34.8)	295 (34.7)	594 (34.8)

Note. CFU, colony-forming units; HC, high concern; LMC, low-to-moderate concern.

### TJF-Q180V culture results

Of the samples collected from TJF-Q180V duodenoscopes, 35 were contaminated with HC organisms (contamination rate, 4.1%) (Table 1). Overall, 38 HC organisms were isolated from 35 samples with the following distributions: 9 gastrointestinal (23.7%), 12 human origin (31.6%), 10 environmental (26.3%), and 7 waterborne (18.4%) (Table 2). We detected no contamination (0 CFU) in 299 samples (34.8%). The 893 LMC organisms were classified into 454 isolates of human origin (50.8%) and 432 isolates of environmental origin (48.4%), excluding 7 unidentified species (0.8%) that could not be classified (Table 3).

**Table 2.** Classification of High-Concern (HC) Organisms Detected by Culture

TJF-Q180V Duodenoscopes			
Classification^a^	HC Organism Detected on Culture	No of Duodenoscopes with This HC Organism	Ratio of the Classified HC Organism
Gastrointestinal	*Klebsiella* spp	3	9/38 (23.7%)
	*Enterococcus* spp	2	
	*Escherichia* spp	2	
	*Pantoea* spp	1	
	*Pluralibicter pyrinus*	1	
Human-origin (other than Gastrointestinal)	*Staphylococcus lugdunensis*	5	12/38(31.6%)
	*Staphylococcus aureus*	5	
	*Candida* spp^a^	2	
Environmental	*Acinetobacter* spp	4	10/38 (26.3%)
	*Candida* spp^a^	1	
	*Erwinia billingae*	1	
	*Pseudoxanthomonas* spp	1	
	*Ralstonia* spp	1	
	*Roseomonas* spp	1	
	*Sphingomonas mucosissima*	1	
Waterborne	*Brevundimonas* spp	3	7/38 (18.4%)
	*Massilia* spp	2	
	*Pseudomonas* spp	2	
TJF-160F/VF Duodenoscopes			
Classification^a^	HC Organism Detected on Culture	No. of Duodenoscopes with This HC Organism	Ratio of the Classified HC Organism
Gastrointestinal	*Klebsiella* spp	14	40/70 (57.1%)
	*Enterococcus* spp	14	
	*Escherichia* spp	6	
	*Enterobacter* spp	3	
	*Raoultella* spp	1	
	*Leclercia* spp	1	
	*Citrobacter* spp	1	
Human origin (other than gastrointestinal)	*Staphylococcus aureus*	2	6/70 (8.6%)
	*Candida* spp^a^	2	
	*Staphylococcus lugdunensis*	1	
	β-hemolytic *Streptococcus* spp	1	
Environmental	*Candida* spp^a^	5	16/70 (22.9%)
	*Acinetobacter* spp	4	
	*Roseomonas* spp	3	
	*Serratia* spp	2	
	*Lysobacter* spp	1	
	*Cupriavidus* spp	1	
Waterborne	*Pseudomonas* spp	4	7/70 (10.0%)
	*Brevundimonas* spp	1	
	*Massilia* spp	1	
	*Achromobacter* spp	1	
Not classified	Gram-negative rod	1	1/70 (1.4%)

Note. Some organisms can have multiple sources but were classified in only one “most probable” category. *Candida* spp was classified as either human origin or environmental depending on the other investigations, including the nature of the concomitant low- to moderate-concern organisms.

a Classification: HC organisms were categorized into the 4 categories based on “most probable” source.

**Table 3.** Classification of Low- to Moderate-Concern (LMC) Organisms Detected by Culture

TJF-Q180V Duodenoscopes			
Classification^a^	Detected LCM Organisms	No. of Duodenoscopes with This Organism	Ratio of the Classified LMC Organisms
Environmental	*Bacillus* spp	264	432/893 (48.4%)
	*Paenibacillus* spp	60	
	*Lysinibacillus* spp	11	
	*Kytococcus* spp	10	
	*Microbacterium* spp	8	
	*Brevibacterium* spp	7	
	*Citricoccus* spp	6	
	Other organisms	66	
Human origin (other than Gastrointestinal)	*Micrococcus* spp	182	454/893 (50.8%)
	*Staphylococcus* spp	147	
	*Corynebacterium* spp	59	
	*Kocuria* spp	28	
	*Rothia* spp	9	
	*Actinomyces* spp	6	
	*Dermacoccus* spp	6	
	Other organisms	17	
Not classified	Unknown	7	7/893 (0.8%)
TJF-160F/VF Duodenoscopes			
Classification^a^	Detected LCM Organisms	No. of Duodenoscopes with This Organism	Ratio of tde Classified LMC Organisms
Environmental	*Bacillus* spp	242	371/752 (49.3%)
	*Paenibacillus* spp	54	
	*Lysinibacillus* spp	18	
	*Kroppenstedtia* spp	10	
	*Microbacterium* spp	8	
	*Brevibacterium* spp	6	
	Other organisms	33	
Human origin (other than gastrointestinal)	*Micrococcus* spp	150	380/752 (50.6%)
	*Staphylococcus* spp	146	
	*Corynebacterium* spp	32	
	*Kocuria* spp	13	
	*Rothia* spp	11	
	*Actinomyces* spp	5	
	Other organisms	18	
Not classified	Unknown	1	1/752 (0.1%)

Note. some organisms can have multiple sources but were classified in only 1 “most probable” category (eg, *Actinomyces*, etc).

a Classification: Low- to moderate-concern organisms were categorized into the 2 categories based on “most probable” source.

### TJF-160F/VF culture results

Of the samples collected from TJF-160F/VF duodenoscopes, 56 (6.6%) were contaminated with HC organisms. Overall, 70 HC organisms were isolated from 56 samples (Table 1) with the following distributions: 40 (57.1%) were gastrointestinal, 6 (8.6%) were classified as human origin, 16 (22.9%) were classified as environmental, 7 (10%) were waterborne, and 1 (1.4%) could not be classified (Table 2). We detected no contamination in 295 samples (34.7%). Of the 752 LMC organisms, 380 (50.5%) were classified as human origin and 371 (49.3%) were classified as environmental, excluding 1 unidentified species (0.1%) that could not be classified (Table 3).

### Distribution of actionable and nonactionable culture results

**Fig. 1.**
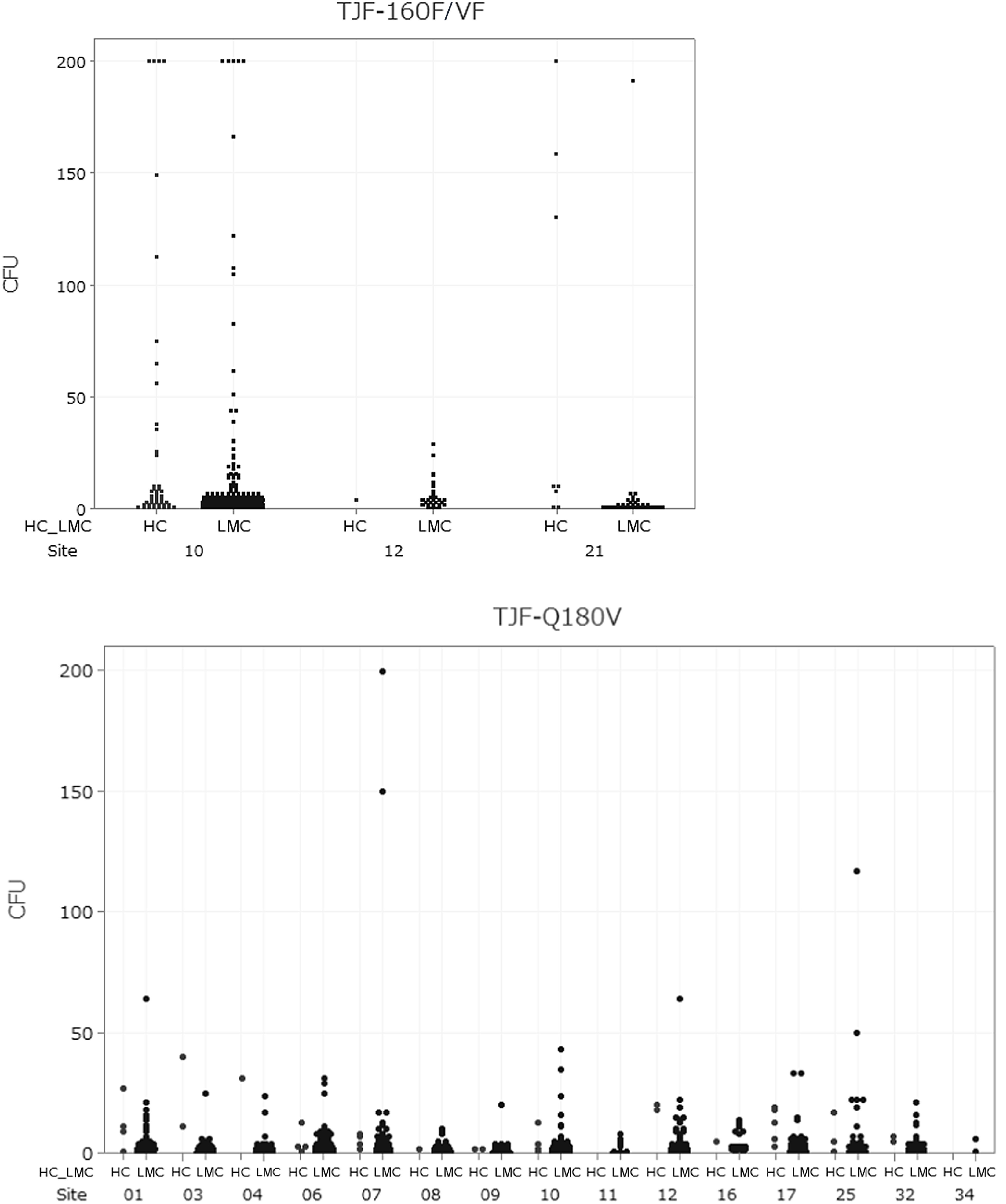
Distribution of colony-forming units (CFU) for each site for all high-concern organisms or >100 CFU of low to moderate concern (HC) and ≤100 CFU of low- to moderate-concern organisms (LMC). Note. Y-axes scale up to 200 CFU along with 1 upper bound demonstrating >200 CFU.

Figure 1 displays the comparison between actionable and nonactionable CFU (ie, no HC organisms and ≤100 CFU of LMC organisms), illustrating the distribution pattern of positive culture results. Many culture-positive TJF-Q180V and TJF-160F/VF duodenoscope samples contained LMC organisms detected at nonactionable levels (data not presented). Additionally, LMC organisms were frequently detected alongside HC organisms in duodenoscope cultures.

### Review of sampling procedure, environmental culture, and negative controls

A retrospective reprocessing review was conducted for a subset of 94 samples (32, TJF-Q180V; 62, TJF-160F/VF), identifying improper reprocessing in 26 samples (27.7%). Sampling was reviewed for a subset of 101 samples (TJF-Q180V, 38; TJF-160F/VF, 63), recording improper sampling in 42 samples (41.6%).

After duodenoscope sampling, environmental sampling was conducted at some collection sites. We analyzed 151 environmental samples from 20 sampling areas at 7 sites to assess the types of organisms in the site environment and their potential impact on duodenoscope contamination (Table 4).

**Table 4.** High-Concern Organisms Isolated During Environmental Sampling and Their Sampling Points

Isolated Organism	Classification	Sampling Point
*Pantoea* spp^a^	Gastrointestinal	Floor Floor mat in front of sink for leakage test Air sample collected using open petri dish
*Staphylococcus* spp^a,b^	Human origin	Floor mat in front of the sink Floor in front of AER
*Acinetobacter* spp^a^	Environmental	Floor in front of storage cabinet Floor mat in front of sink of manual cleaning
*Azospirillum* spp		Bottom of sink for manual cleaning
*Chryseobacterium* spp		Bottom of sink for manual cleaning
*Ochrobactrum* spp		Floor mat in front of sink for manual cleaning
*Phenylobacterium* spp		Floor mat in front of sink for manual cleaning
*Roseomonas* spp^a^		Floor Floor mat in front of sink
*Pseudomonas* spp^a^	Waterborne	Floor Floor in front of AER Bottom of sink for manual cleaning
*Blastomonas* spp		Final rinse water from AER

Note. AER, automated endoscope reprocessor.

a These HC organisms were also isolated from clinically used duodenoscopes.

b *Staphylococcus aureus* or *S. lugdunensis.*

Negative control samples were collected from duodenoscopes that underwent EtO-sterilization and subsequent AER reprocessing. Of the 59 samples collected at 6 sites, 1 contained an HC organism (1.7%) and 12 were not contaminated (20.3%). We also sampled EtO-sterilized duodenoscopes without further AER reprocessing. Of the 25 samples collected at 3 sites, 4 samples were not contaminated (16.0%). The LMC and HC organisms in the negative controls are listed in Table 5.

**Table 5.** Culture Results From Duodenoscopes Used as Negative Controls

Cultures Collected	Clinically Used Duodenoscopes Sterilized with EtO Followed by AER Cycle (n=59), No. (%)^a^	Clinically Used Duodenoscopes Sterilized With EtO Only (n=25), No. (%)^b^
HC organisms^c^	1 (1.7)	0 (0)
>100 CFU LMC organisms	2 (3.4)	1 (4.0)
11–100 CFU of LMC organisms	4 (6.8)	4 (16.0)
1–10 CFU of LMC organisms	40 (67.8)	16 (64.0)
0 CFU no contamination	12 (20.3)	4 (16.0)

Note. EtO, ethylene oxide; AER, automated endoscope reprocessor; HC, high concern; LMC, low-to-moderate concern.

a These duodenoscopes had been used in the study on patients and had been fully reprocessed with EtO sterilization as the final step. These duodenoscopes were processed through the onsite AER before taking samples for culture. The same process were as used at sites that sent duodenoscopes for EtO sterilization after clinical use and reprocessing. The patient-used duodenoscopes that were sent for EtO sterilization were subsequently processed through an AER then put into storage cabinets before the next clinical use.

b Duodenoscopes that had been used in the study on patients and had been fully reprocessed with EtO sterilization as the final step. These duodenoscopes were not processed through an AER before taking samples for culture.

c *Pseudomonas luteola* was detected.

## Discussion

In this novel, multicenter, real-world clinical study, we used validated and sensitive culture methodology to assess the level of bacterial contamination in reprocessed duodenoscopes. We investigated the contamination rates of TJF-Q180V and TJF-160F/VF duodenoscopes following clinical use and reprocessing, revealing the duodenoscope contamination rates in a “nonoutbreak” setting in the United States.

Overall, 5.3% of reprocessed duodenoscopes were contaminated with HC organisms (TFJ-Q180V, 4.1%; TJF-160F/VF, 6.6%) and 0.8% were contaminated with >100 CFU LMC organisms (TFJ-Q180V, 0.3%; TJF-160F/VF, 1.2%). Of the 16 evaluated collection sites, 15 had some HC contamination, suggesting a substantial rate of possible occurrence at large and small endoscopy facilities.

Previously reported contamination rates vary greatly, with studies reporting overall rates of 0.4%–30%^9–14^ and HC or action-level contamination rates of 0.2%–15%.^10–14^ In our study, the HC contamination rate (5.3%) was lower than the 15% rate reported by Rauwers et al in 2018^13^ and 2020^14^ but was higher than the rates reported by other studies. We concluded that the differences in duodenoscope sampling and culture methodologies, definitions of HC organisms, and broad classification criteria for actionable CFU levels of LMC organisms might explain the wide-ranging contamination rates.

A 5-year review^9^ reported an HC contamination rate of 0.4%, based on which the FDA designed the current PMS series.^23^ Gillespie et al^9^ flushed the channels with 10 mL of sterile water, centrifuged and concentrated the samples, and cultured only 0.1 mL of the reconstituted 1-mL fluid for CFU assessment, allowing organisms with a concentration of ≥10 CFU/mL in a sample to be detected (provided all bacteria initially present in the 10 ml were concentrated in the 1-mL fluid and evenly distributed in the concentrated sample). The method of Gillespie et al revealed an HC concentration of ≥10 CFU in 18 samples, resulting in a 1.1% HC contamination rate.

In contrast, Rauwers et al^13^ reported a 15.3% HC contamination rate (≥1 CFU/20 mL of microorganisms of gastrointestinal or oral origin).

Similar to our method, their sampling protocol consisted of flushing and brushing the biopsy and suction channels and swabbing the forceps elevator with the sample concentrated using filtration. However, the inclusion of bacteria of oral origin because HC organisms differ from the FDA–CDC–ASM protocol criteria^22^ and may explain the higher rate of actionable organisms in their study.^13^ Furthermore, their study was a multicenter study that involved 73 sites with no surveillance on protocol adherence during reprocessing or sampling, which may be another reason for the high contamination rate. Future literature reviews need to consider the different sampling and interpretation methodologies used when comparing contamination rates.

We conducted environmental sampling to evaluate the regular environmental condition from 7 collection sites after duodenoscope sampling. We classified HC organisms into gastrointestinal, human origin, environmental, and waterborne types (Table 4). Interestingly, we isolated *Pantoea* spp, which is closely related to *Enterobacteriaceae* and infects humans from the environment. We determined that 5 of the 8 HC organisms detected in the environmental cultures were also isolated from clinically used duodenoscopes. Although the environment may be contaminated by a nondisinfected endoscope, these results suggest that accidental environmental contamination of the endoscope may occur if aseptic techniques are not followed and/or if the environment is insufficiently disinfected before sampling.

If duodenoscopes are properly disinfected, but the environment is contaminated, the scopes may become contaminated after reprocessing during handling without gloves, storage with moisture in channels, and/or during endoscope sampling. The presence of 1–10 CFU, which was frequently seen in both negative controls (>64%) as well as patient-used, fully reprocessed duodenoscopes (56%), suggests that the contamination for most LMC organisms detected by culture may be mainly due to accidental environmental contamination during or before sampling. The instances in which >100 CFU LMC organisms were detected in nonclinically and clinically used scopes suggest that the endoscope may have also been contaminated with a replicable environmental organism (possibly during storage). Ensuring dry storage is crucial to preventing biofilm formation in endoscope channels during storage.^24^

When we detected HC organisms or >100 CFU of LMC organisms, we reviewed the sampling and reprocessing to identify improper sample handling. In ∼41.6% of the reviewed samples, improper handling because of inappropriate personal protective equipment or improper aseptic techniques was detected, which may have caused accidental environmental contamination of reprocessed endoscopes.

Eliminating low levels (eg, 11–100 CFU) of environmental contamination with LMC organisms during endoscope channel sampling can be difficult. We recommend that routine duodenoscope sampling is continued to establish a baseline of LMC environmental contamination levels per site. If the contamination rate exceeds the baseline, the reprocessing procedure should be inspected despite not reaching an actionable level >100 CFU. Hospitals should continue to maintain clean and/or disinfected environments and should establish ongoing training workshops for sampling technicians to reduce environmental contamination.

Historically, most endoscope-transmitted infections have resulted from reprocessing errors, commonly human error.^25^ We observed deviations from correct duodenoscopy-specific reprocessing steps (eg, not flushing the elevator recess and not raising or lowering the forceps elevator during precleaning or manual cleaning) in 27.7% of the procedural reviews. To reduce improper reprocessing, we recommend that the instructions for the use of reprocessing are routinely reviewed for further updates. The healthcare reprocessing staff should be offered additional ongoing training (if required) and assistance training (when staff turnover is high).

Disposable duodenoscopes have recently been commercialized. In August 2019, the FDA recommended their use “to reduce risk of patient infection.”^26^ Although disposal may address duodenoscope-transmitted infections, the cost of disposable parts will be markedly high, and the large amount of annual medical waste generated will negatively impact the environment. Instead, the environmental impact should be minimized.^27,28^ Disposable duodenoscopes may not be as functionally effective and safe as well-established reusable ones. The risks of procedural failures due to disposable scopes may be greater than the low risk of a duodenoscope-transmitted infection.^27,29^ Development and use of sterilizable scopes could be one of the solutions to reduce medical waste and contamination risk.

Our study data provide insight into the current duodenoscope reprocessing procedures at healthcare facilities across the United States in a “nonoutbreak” setting. We utilized validated, sensitive culture methodologies^22^ to evaluate the contamination rate. We confirmed that HC organisms could be recovered inside duodenoscope channels and lever reprocessed recess areas. HC contamination of reprocessed duodenoscopes does not directly lead to infection. Although the relationship between endoscopic contamination and infection occurrence remains unclear, instructions for use for reprocessing should be improved to make it easier for staff to understand and/or follow, and human errors during reprocessing should be addressed. Proffer of an off-site training module for reprocessing including online training will be needed. The analysis and correction of endoscope contamination may reduce or eliminate HC contamination. Moreover, the design of the duodenoscope distal end and additional design changes facilitating endoscope cleaning and disinfecting should be considered. Hospitals should continue stringent aseptic protocols, maintain clean environments, and conduct ongoing staff training programs. Improving sampling and culture methods during duodenoscope surveillance may also reduce environmental contamination during sampling.
